# A Multi-Layer-Controlled Strategy for Cloning and Expression of Toxin Genes in *Escherichia coli*

**DOI:** 10.3390/toxins15080508

**Published:** 2023-08-18

**Authors:** Jessie Vandierendonck, Yana Girardin, Pieter De Bruyn, Henri De Greve, Remy Loris

**Affiliations:** Center for Structural Biology, Vlaams Instituut voor Biotechnologie and Structural Biology Brussels, Vrije Universiteit Brussel, Pleinlaan 2, B-1050 Brussel, Belgium; jessie.vandierendonck@vub.be (J.V.); yana.andrea.girardin@vub.be (Y.G.);

**Keywords:** toxins, cloning, riboswitch, transcriptional control, translational control, replicational control

## Abstract

Molecular cloning and controlled expression remain challenging when the target gene encodes a protein that is toxic to the host. We developed a set of multi-layer control systems to enable cloning of genes encoding proteins known to be highly toxic in *Escherichia coli* and other bacteria. The different multi-layer control systems combine a promoter–operator system on a transcriptional level with a riboswitch for translational control. Additionally, replicational control is ensured by using a strain that reduces the plasmid copy number. The use of weaker promoters (such as P*_BAD_* or P*fdeA*) in combination with the effective theophylline riboswitch is essential for cloning genes that encode notoriously toxic proteins that directly target translation and transcription. Controlled overexpression is possible, allowing the system to be used for evaluating in vivo effects of the toxin. Systems with a stronger promoter can be used for successful overexpression and purification of the desired protein but are limited to toxins that are more moderate and do not interfere with their own production.

## 1. Introduction

Cloning and production of recombinant proteins are well-established techniques in molecular biology. They are routinely carried out in a variety of both prokaryotic and eukaryotic hosts. *Escherichia coli* (*E. coli*) is often the preferred host organism because of its short generation time of 20 min, high ability of exogeneous DNA uptake and efficient protein expression [1,2,3]. Still, cloning of genes encoding proteins that interfere with bacterial growth or otherwise exhibit toxic effects often remains challenging.

One way to circumvent toxic effects is by using bacterial strains that are resistant to a particular toxin. This has for example been proven successful for toxins CcdB and VapC, where efficient cloning and production of the wild-type toxins was possible [4,5]. Unfortunately, such resistant strains are more than often not available. In particular, endoribonucleases pose problems as it is unfeasible to mutate their RNA targets. Moreover, the development of a suitable resistant strain can be time-consuming as it requires prior insight into the toxin–target relationship.

A different way to manage cloning of weakly toxic genes is to lower the copy number of their harboring plasmid. Regulation of plasmid replication can be accomplished by using low copy-number vectors that tightly regulate the toxin’s expression, a well-known example being the pBAD vectors [6]. Alternatively, one can consider the use of low copy number strains such as the Copycutter^TM^ EPI400^TM^ *E. coli* strain, which is optimized for cloning toxic genes. Such strains reduce the copy number of pUC- or pET-vectors to only 10 copies per cell, but upon induction, the copy number will increase significantly, resulting in higher yields of toxin protein [7].

However, even with low plasmid copy numbers, leaky expression might still pose problems and needs to be suppressed. This can be achieved on both the transcriptional and/or translational levels. Most gene expression control systems are solely based on transcriptional regulation. The use of an inducible promoter regulates transcription of the downstream gene(s) stringently in time and space. Common examples of chemically inducible promoters are the strong IPTG-inducible P*lac* or its stronger derivative P*tac* and the weaker arabinose-inducible P*_BAD_*, where expression can be repressed by glucose, reducing leaky transcription [6,8,9]. In practice, however, such systems are not tight ON/OFF switches and leaky expression is almost inevitable. Besides chemical inducers, temperature-inducible promoters known for limited leakiness also exist. However, there is often a stress burden linked to the shifts in temperature [10]. The promoter region can also be mutated as such to reduce its activity and thus leaky expression as described by [11]. There, a two-thymidine deletion in the −35 region of the T5 promoter led to the successful cloning of the *ϕ*174 lysis gene E. As a last resort, site-specific point mutations in the toxin gene that render the toxin harmless for the cell can be introduced to enable the cloning and overexpression of the non-toxic protein. Examples of such non-toxic mutants are *E. coli* MazF^E24A^ (*Ec*MazF^E24A^) or bacteriophage P1 Doc^H66Y^ (*P1*Doc^H66Y^) [12,13]. Nonetheless, mutating the toxin often results in loss of functionality, limiting the usefulness of downstream experiments severely.

In addition to transcriptional control, regulation at the translational level is described in literature. These include ribozymes that are activated by bound ligands enabling site-specific digestion of RNA to control gene expression, antisense RNAs or the incorporation of site-specific unnatural amino acids [14,15,16,17]. Another well-known translational control element is the riboswitch. This is an RNA element that functions by regulating protein expression in response to the presence of a certain metabolite. Riboswitches consist of two domains, called the aptamer and the expression platform. The aptamer domain selectively binds a metabolite, thereby causing the formation of a terminator structure in the expression platform. This conformational change in RNA structure controls the expression of the downstream gene [18]. Different riboswitches have been discovered, engineered or synthesized in the past. Examples are the adenine riboswitch, vitamin B_12_ riboswitch, theophylline riboswitch and many more [19,20,21].

Altogether, for all the strategies described above, several disadvantages remain. Controlling expression on either the transcriptional or the translational level often seems insufficient to avoid leaky expression of highly toxic genes. Therefore, a combination of both is frequently desired. Combined transcription–translation regulating systems have already been developed and reported in the past (for a review, see [22]). A dual-control system named *RiboTite*, using inducible promoters and a riboswitch, was reported by Morra et al. [23]. Other authors make use of antisense RNA (asRNA) regulators that are configured in such a way that it targets both transcriptional and translational regulation to increase the control over gene expression [15]. Lee et al. furthermore discussed the combined use of asRNA and small transcription-activating RNA for deactivation or activation of gene expression, respectively [24]. The use of asRNA has also been combined with the use of a strong repressible promoter [25]. Additionally, dual control over gene expression has been achieved by constructing tandem theophylline riboswitches and the RepA protein degradation tag, targeting both transcription and post-translational regulation [26].

Among the most difficult toxic genes to clone are those encoded by bacterial toxin–antitoxin (TA) systems. The toxins on their own can almost never be cloned without introducing mutations; even cloning the operon, including both toxin and antitoxin genes, can sometimes be cumbersome, e.g., for the *phd/doc* homolog *tasAB* [27]. Here, we describe and evaluate a novel multi-control system where TA toxins are cloned and expressed in the absence of their cognate antitoxin. This model system inhibits leaky expression on various levels: (1) on the replicational level using a strain that reduces the plasmid copy number, (2) on the transcriptional level by a tightly controllable inducible promoter and (3) on the translational level using a ligand-dependent riboswitch.

## 2. Results and Discussion

### 2.1. Choice of Toxins

The initial motivation for developing this cloning strategy was our search to clone the extremely potent parE2 toxin gene from Vibrio cholerae. To demonstrate the broader applicability of our multi-control system, we selected a set of seven toxins with distinct activities and levels of toxicity to evaluate its usefulness for cloning toxins. Six out of the seven toxins are part of a toxin–antitoxin system: *Escherichia coli* MazF (*Ec*MazF), *Vibrio cholerae* HigB2 (*Vc*HigB2) bacteriophage P1 Doc (*P1*Doc), *E. coli* O157 and *V. cholerae* ParE2 (*Ec*ParE2 and *Vc*ParE2) and F-plasmid CcdB (*F*CcdB). These toxins represent three different activities: ribonuclease (RNase), translation-inhibiting kinase and gyrase poisoning activity. MazF toxins are endoribonucleases (RNases) that cut mRNA at 5′ ACA sites, while HigB2 toxins are ribosome-dependent RNases that cut mRNA, which is actively being translated [28,29]. Doc inhibits translation by phosphorylating elongation factor Tu, and CcdB and ParE2 toxins interfere with transcription and replication by poisoning gyrase [30,31,32,33]. In addition, two non-toxic point mutants were added as controls: H66Y for *P1*Doc and E24A for *Ec*MazF. These point mutants can be overexpressed in the absence of their cognate antitoxin and produced with high yields [12,13]. Finally, barnase was included in our screen. This scavenging ribonuclease from *Bacillus amyloliquefaciens* is exported but gets protected via its interaction with its inhibitor barstar while inside the cell [34].

### 2.2. Design of Different Multi-Control Systems

**Figure 1:**
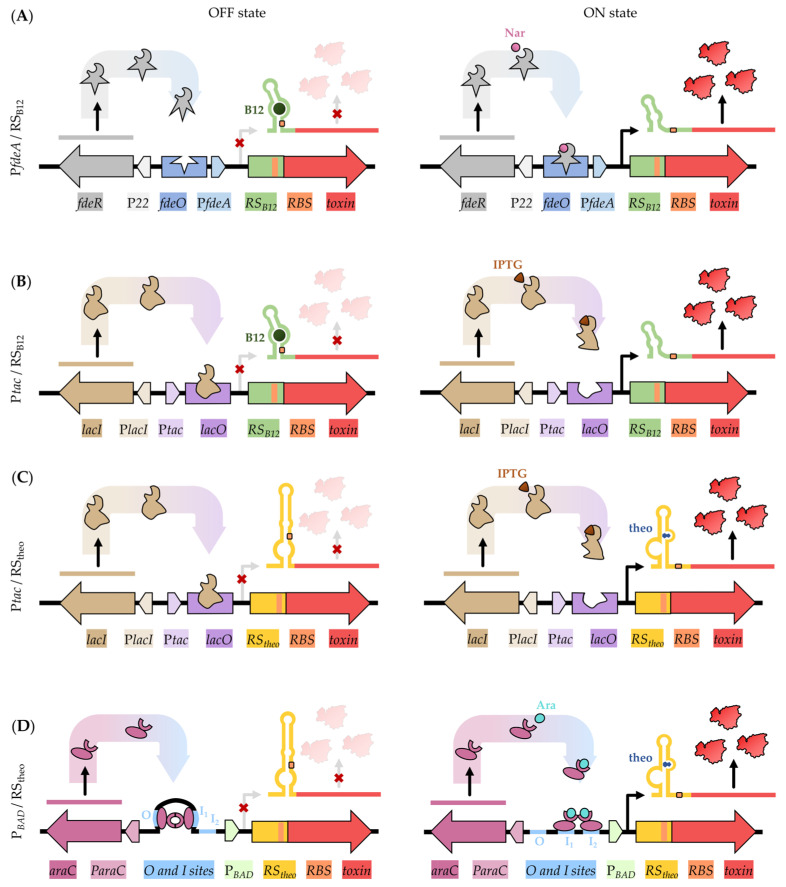
Schematic overview of transcriptional and translational layers of control for the four systems developed in this study. Different promoters and riboswitches were combined to control the toxin’s (red) gene expression. (**A**) P*fdeA*-RS_B12_ combination. The naringenin-inducible promoter P*fdeA* in light blue is positively regulated by the FdeR activator (grey) in presence of naringenin and is combined with the negatively regulated vitamin B_12_ riboswitch (RS_B12,_ green). Only in absence of vitamin B_12_, the ribosome binding site (RBS, orange) is accessible for ribosome binding. (**B**) P*tac*-RS_B12_ combination. The IPTG-inducible tac promoter (P*tac*) in light purple is repressed by binding of the LacI repressor (brown) to the *lac* operator (*lacO*, purple) in absence of IPTG. Presence of IPTG allows transcription initiation. This is combined with the B_12_ riboswitch. (**C**) P*tac*-RS_theo_ combination. The *lacI/lacO* operator system is combined with the positively regulated synthetic theophylline riboswitch (RS_theo_, yellow). Presence of theophylline (blue) alters the riboswitch conformation, allowing access to the RBS and therefore translation. (**D**) P*_BAD_*-RS_theo_ combination. The arabinose-inducible promoter P*_BAD_* (light green) is positively regulated through the binding of the AraC activator (magenta) to the I1 and I2 sites when bound to arabinose (turquoise). Combination with the RS_theo_ allows tight regulation of the toxic gene.

Two different riboswitches were evaluated for their ability to control translation tightly: a vitamin B_12_ riboswitch and a synthetic theophylline riboswitch. The vitamin B_12_ riboswitch originates from the *btuB* gene encoding the BtuB receptor protein located in the outer membrane of *E. coli* [20]. When vitamin B_12_ is present, it binds to the aptamer domain of the riboswitch (Figure 1A,B). This results in a conformation where the ribosome binding site (RBS) on the transcript is shielded, causing translational repression (OFF-state). Once vitamin B_12_ is removed, translation is initiated and ribosomal access to the RBS is restored (ON-state). The synthetic theophylline riboswitch was initially created for tightly regulated expression of chloroplast genes [21]. It works in the opposite way compared to vitamin B_12_: the presence of theophylline ensures translation of the downstream gene(s) as the RBS is accessible (Figure 1C,D).

To add a transcriptional layer of control, the riboswitches described above were combined with different promoter–operator systems. In the first system (Figure 1A), the vitamin B_12_ riboswitch was combined with the naringenin-inducible promoter P*fdeA* to regulate gene expression on a transcriptional level [35]. In the presence of naringenin, the transcriptional activator FdeR binds to the operator site *fdeO*, thereby initiating transcription.

As the P*fdeA* promoter is rather weak for overexpression of the toxin, a second system was designed where P*fdeA* was exchanged for the stronger IPTG-inducible *tac* promoter (P*tac*) (Figure 1B). This hybrid promoter combines the −35 region of the *trp* promoter with the −10 region of the lacUV5 promoter–operator region [9]. As P*tac* is a strong promoter, it is mainly of interest when aiming for protein production. Similar to P*fdeA*, P*tac* is a negatively inducible promoter that is controlled by the inactivation of the LacI repressor via allolactose or its analog isopropyl β-D-1-thiogalactopyranoside (IPTG). The presence of the latter compounds changes the conformation of the LacI repressor, which restrains its binding to the *lacO* operator and consequently allows transcription. In order to compare the efficiency of both the B_12_ and theophylline riboswitches, a similar system combining P*tac* with the theophylline riboswitch was designed (Figure 1C).

Finally, the tightly regulated arabinose-inducible promoter (P*_BAD_*) was included in this study in combination with the theophylline riboswitch (Figure 1D). P*_BAD_* is regulated by the activator AraC. In the absence of arabinose, AraC binds to the O and I1 sites, thereby preventing transcription. Only when arabinose is bound to AraC, transcription is initiated as AraC binds to the I1 and I2 sites.

As an extra layer of control, all systems were inserted in CopyCutter *E. coli* EPI400 cells (LGC Biosearch Technologies, Teddington, England). These cells have the ability to lower the plasmid copy number, which helps to reduce leaky expression levels of the toxic genes. This extra layer showed to be relevant, as transformation of toxin-carrying systems in *E. coli* NEB5*α* cells did not result in any transformants. The use of this strain has the additional advantage that the same plasmid can be used for both cloning and expression purposes. While non-induced, the low copy number helps for successful cloning of the more problematic toxins, but at the same time, protein production can be attempted after inducing the copy number without the need to reclone the toxin in a higher copy number plasmid (which may be problematic).

All three different layers of control mentioned above were combined to increase cloning efficiency. When gene expression is desired, the toxin production can be induced on transcriptional, translational and replicational levels.

### 2.3. Cloning Efficiency of the Different Systems

Regulation through the use of both the naringenin-inducible P*fdeA* promoter and the vitamin B_12_ riboswitch led to successful cloning of most of the toxic genes: for all toxins except the highly toxic *Vc*ParE2, mutation-free transformants were obtained in *E. coli* CopyCutter EPI400 cells (Table 1). This was confirmed by colony PCR and Sanger sequencing. 

The stronger tac promoter (P*tac*) was combined with the vitamin B_12_ riboswitch to evaluate the effect of producing more toxin proteins per cell. Here, only clones of non-toxic mutants and the weak toxin *Ec*MazF could be obtained.

However, inserting all toxins in a system harboring P*tac* and the theophylline riboswitch appeared to be more successful. For this system, a combination of mutation-free toxins (*Ec*MazF, *Ec*ParE2, *Vc*HigB2) and toxins that carry point mutations, albeit still significantly toxic (*P1*Doc, *F*CcdB_,_ barnase), as shown later, were successfully cloned. These mutations include Doc N69H and the double mutant A32T/A63V. For CcdB, we recovered the S22I point mutant and for barnase either I52T or S93R. Later, the point mutants Doc^N69H^, CcdB^S22I^ and barnase^I52T^ will be used for further experiments on this system. From now on, we will refer to them as *P1*Doc*, *F*CcdB* and barnase*.

The higher success rate for cloning toxic genes in the P*tac*—RS_theo_ system compared to P*tac*—RS_B12_ indicates that the theophylline riboswitch suppresses translation in a more efficient manner than the vitamin B_12_ riboswitch.

Cloning of *Vc*ParE2 failed for all the above strategies, as frameshift mutations were repeatedly encountered. Therefore, the *Vc*ParE2 gene was inserted in a system combining the efficient theophylline riboswitch with the tighter controlled and weaker arabinose-inducible promoter (P*_BAD_*). This ultimately led to the successful cloning of a mutation-free *Vc*ParE2 in the absence of its cognate ParD2 antitoxin.

### 2.4. Assessing Toxicity upon Inducing Gene Expression

#### 2.4.1. Combination of the P*fdeA* Promoter with the RS_B12_ Riboswitch

**Figure 2:**
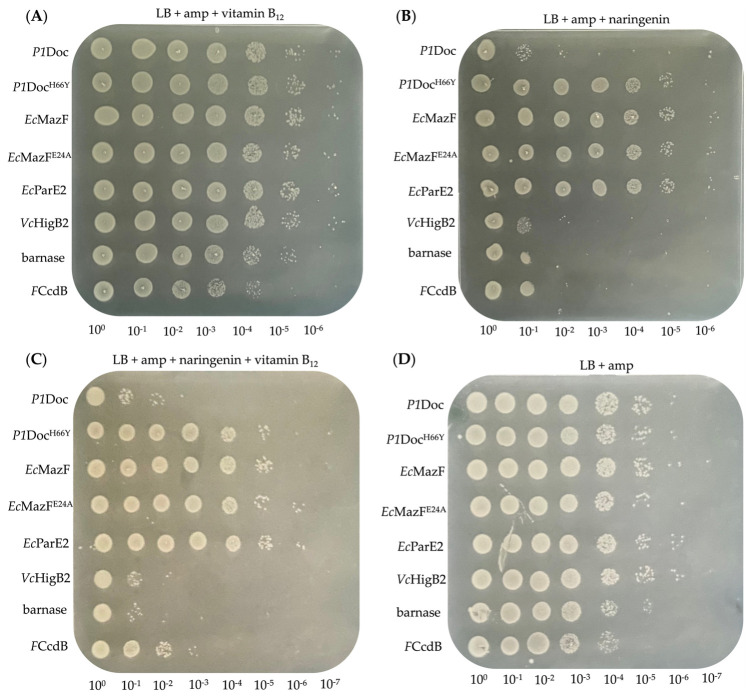
Spot test of serial diluted toxins on plates with or without inducing agents for the P*fdeA*—RS_B12_ strategy. *E. coli* EPI400 harboring the successfully cloned toxins *P1*Doc, *P1*Doc^H66Y^, *Ec*MazF, *Ec*MazF^E24A^, *Ec*ParE2, *Vc*HigB2, barnase and FCcdB on the pJYP1 vector was spotted on LB agar plates supplemented with ampicillin and additionally vitamin B_12_ (**A**, OFF-state) or naringenin (**B**, ON-state) or a combination of both (**C**, partly induced) or none of the additional agents (**D**, partly induced).

**Figure 3:**
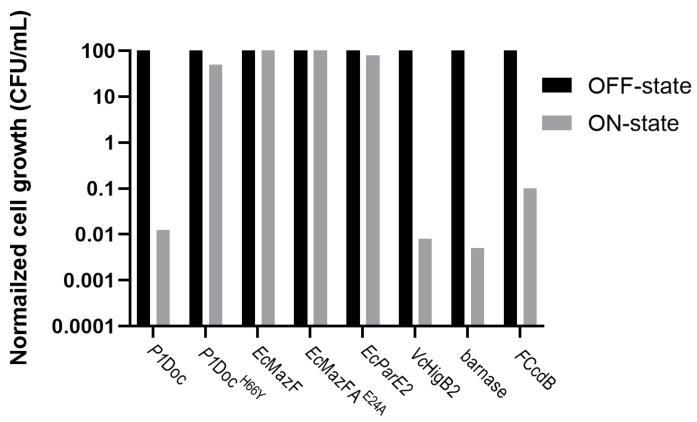
Logarithmic normalized overview of cell growth observed for the P*fdeA*-RS_B12_ strategy. Colonies are counted from the spot test on different LB ampicillin plates for *E. coli* EPI400 harboring toxins *P1*Doc, *P1*Doc^H66Y^, *Ec*MazF, *Ec*MazF^E24A^, *Ec*ParE2, *Vc*HigB2, barnase and *F*CcdB. Raw colony count data were normalized to fraction survival for comparison of the OFF-state (vitamin B_12_ and no naringenin, black bars) to the ON-state (naringenin and no vitamin B_12_, grey bars).

During the OFF-state (Figure 2A, black bars on Figure 3), where vitamin B_12_ was added to the plates and gene expression was inhibited, the growth of all toxin-harboring cultures was around 10^9^ CFU/mL. This is also what we would expect for overnight-grown *E. coli* in optimal growth conditions [36]. This indicates that growth when gene expression of the toxins is not induced reaches the optimal conditions. Inducing gene expression (Figure 2B, grey bars on Figure 3), by adding naringenin and removing vitamin B_12_ from the plate media, resulted in a detrimental effect on the host growth for most of the toxins (*P1*Doc, *Vc*HigB2, barnase and *F*CcdB). This caused a reduction of growth with a factor of 10^4^ for barnase, *P1*Doc and *Vc*HigB2 and a factor of 10^3^ for *F*CcdB (Table S1). In contrast, no effect on growth was observed upon induction of *Ec*MazF or *Ec*ParE2. For some potent toxins (such as *P1*Doc, *Vc*HigB2, barnase and *F*CcdB), only a few proteins are enough to kill the cell, while for other toxins (such as *Ec*MazF and *Ec*ParE2) overexpression of the toxin appears to be needed to intervene with cell viability. This all depends on the mode of action of the toxins as well as the stoichiometry of interaction between the toxin and its target inside the cell. In case of MazF, the reduced toxic effect can additionally be explained by the production of the MazE antitoxin by the *E. coli* strain, inhibiting MazF’s action. When comparing Figure 2C with Figure 2D, where gene expression was only partly induced, we can conclude that adding naringenin to the plate had a more significant effect on cell growth compared to the removal of vitamin B_12_ from the plate. This suggests the minor role of the vitamin B_12_ riboswitch in this strategy. For controls, *P1*Doc^H66Y^ and *Ec*MazF^E24A^, no reduction in growth was observed over the different plates.

#### 2.4.2. Combination of the P*tac* Promoter with the RS_theo_ Riboswitch

**Figure 4:**
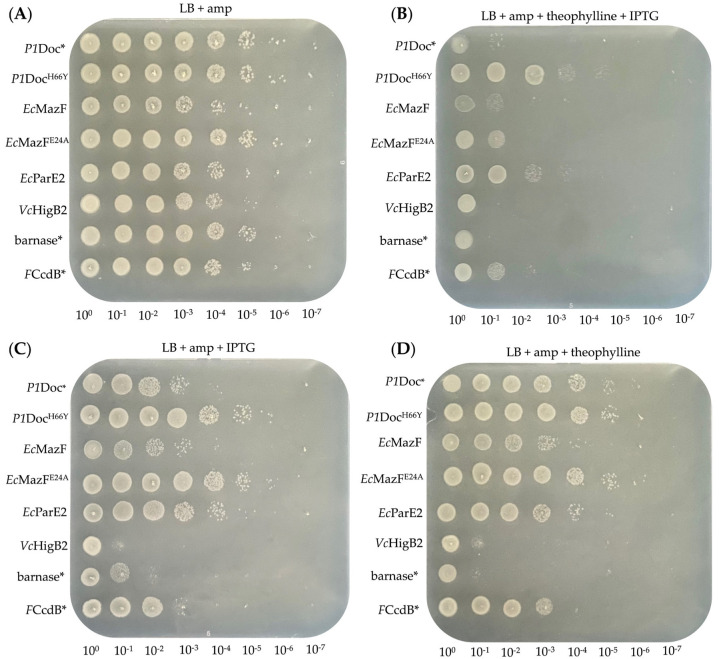
Spot test of serial diluted toxins on plates with or without inducing agents for the P*tac*—RS_theo_ strategy. *E. coli* EPI400 harboring the successfully cloned toxins *P1*Doc*, *P1*Doc^H66Y^, *Ec*MazF, *Ec*MazF^E24A^, *Ec*ParE2, *Vc*HigB2, barnase* and *F*CcdB* on the pJYP3 vector were spotted on LB agar plates supplemented with ampicillin (**A**, OFF-state) and additionally theophylline and IPTG (**B**, ON-state) or IPTG alone (**C**, partly induced) or theophylline alone (**D**, partly induced).

**Figure 5:**
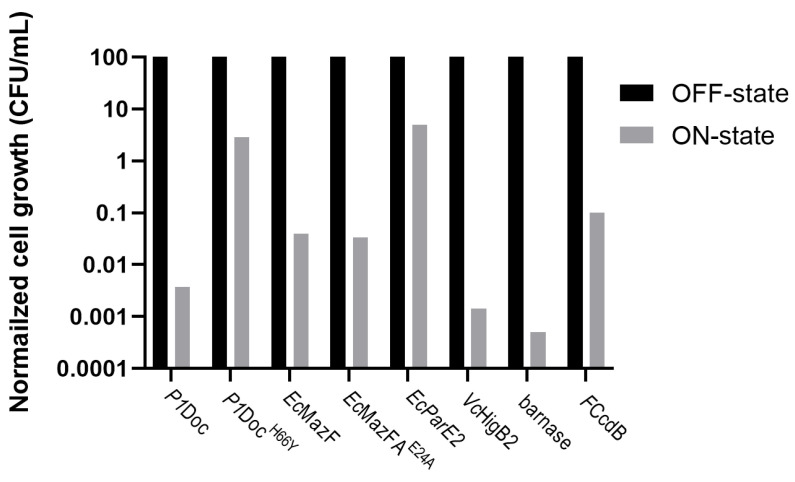
Logarithmic normalized overview of cell growth observed for the P*tac*—RS_theo_ strategy. Colonies are counted from the spot test on different LB ampicillin plates for *E. coli* EPI400 harboring toxins *P1*Doc, *P1*Doc^H66Y^, *Ec*MazF, *Ec*MazF^E24A^, *Ec*ParE2, *Vc*HigB2, barnase and *F*CcdB. Raw colony count data were normalized to fraction survival for comparison of the OFF-state (no theophylline and no IPTG, black bars) to the ON-state (theophylline and IPTG, grey bars).

The same screen was performed for the P*ta*c—theophylline system (Figure 4 and Figure 5), where we observed a dramatic effect on cell growth for all toxins when supplementing inducing agents IPTG and theophylline to the LB ampicillin plates (Figure 4A,B). This decrease in growth corresponds to an average of 10^4^ orders of magnitude for *P1*Doc* and *Ec*MazF, for *P1*Doc^H66Y^ and *Ec*ParE2 only a 10- to 100-fold reduction, for EcMazF^E24A^ and *F*CcdB approximately 3 orders of magnitude and for *Vc*HigB2 and barnase the biggest effect is observed with a 10^5^-fold decrease compared to the OFF-state (Table S2). When comparing the sole effect of IPTG and theophylline, one can conclude that their contribution to growth reduction is notable and comparable (which was not the case for the P*fdeA*-RS_B12_ system). Additionally, they clearly work synergistically as the combined effect of both agents is much higher than when applied separately (Figure 4C,D). In contrast to the P*fdeA*-RS_B12_ system, a clear toxic effect is observed for *Ec*MazF and *Ec*ParE2 when both IPTG and theophylline are combined, most likely because the strong inducible tac promoter ensures higher transcription levels. For the controls, however, a decrease in growth was also observed, with a greater effect for *Ec*MazF^E24A^ than for *P1*Doc^H66Y^. This different observation might be due to the nature of these mutants. While the *P1*Doc^H66Y^ mutant has a point mutation in one of the catalytic residues, the *Ec*MazF^E24A^ mutant is inoperative in recognizing its mRNA substrate sites [13,37]. This mutation increases the cell’s viability drastically compared to wild-type MazF with ten times reduced RNase activity [12]. However, this activity is possibly still high enough once a sufficient number of proteins is produced to impact the host’s survival upon overexpression under the control of the strong P*tac*.

The theophylline riboswitch was shown in the past to tightly control translation of proteins with very low leakiness [26,38]. This agrees with our own result, where the theophylline riboswitch seems to work more stringently than the vitamin B_12_ riboswitch. The system works best in combination with the strong inducible P*tac*. Nevertheless, we must consider that this promoter can have up to 2% leaky expression [8], which might cause difficulties when working with extremely toxic genes.

#### 2.4.3. Combination of the P*_BAD_* Promoter with the RS_theo_ Riboswitch

**Figure 6:**
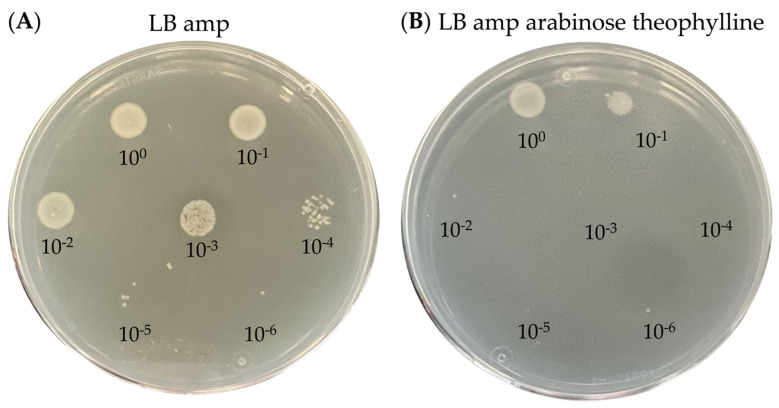
Spot test of serial diluted *E. coli* EPI400 harboring pJYP4_*Vc*ParE2 on plates with or without inducing agents for the P*_BAD_*—RS_theo_ strategy. *E. coli* EPI400 harboring pJYP4_*Vc*ParE2 was spotted on (**A**, OFF-state) LB agar plates supplemented with ampicillin and (**B**, ON-state) theophylline and arabinose.

One way to circumvent this issue of leakiness is by using a weaker inducible promoter, namely P*_BAD_*, having a 20-fold decrease in leaky expression [6]. When combining the theophylline riboswitch with the weaker arabinose promoter, the reduction in growth for *E. coli* EPI400 harboring pJYP4_*Vc*ParE2 upon induction of gene expression was in the range of 10^4^ orders of magnitude (Figure 6). This combination seems to surpass all other systems, as the potent *Vc*ParE2 was successfully cloned only under these stringent conditions.

### 2.5. Applicability of the Ptac—Theophylline System for Protein Production

Next, we wanted to know if our multi-layer control systems also allow for successful overexpression and protein production after induction. We chose the P*tac*—theophylline system as this combines the strongest promoter with the most successful riboswitch. We first evaluated the use of the *E. coli* EPI400 CopyCutter strain to increase the copy number of the expression plasmid upon induction. Depending on the plasmid used, a 2- to 20-fold increase in copy number is typically reported [7]. We therefore determined this increase for pJYP2_doc* and pJYP2_*doc*^H66Y^ via qPCR. In both cases, we observed an approximately 50-fold increase in plasmid copy number upon induction (see Supplementary qPCR results for details: Figure S2 and Tables S3–S6). This not only allows for plasmid preparation at higher yields, but also suggests that useful overexpression may be obtained.

**Figure 7:**
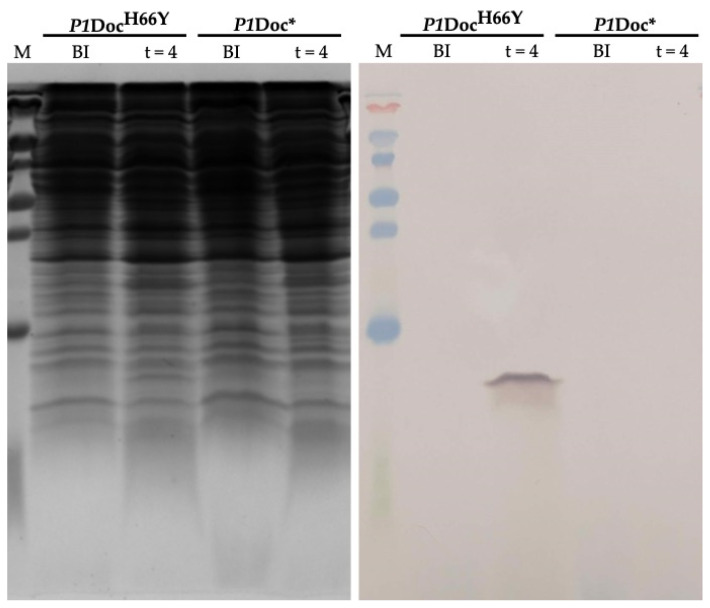
Small-scale expression of *P1*Doc and *P1*Doc^H66Y^ using the P*tac*—RS_theo_ strategy. Samples of *P1*Doc* and *P1*Doc^H66Y^ before induction (BI) and four hours after induction (t = 4) were analyzed via 20% SDS-PAGE (**left**) and anti-histidine Western blot (**right**).

To assess the levels of protein synthesis in the P*tac*—theophylline system, small-scale expression tests were performed for C-terminally histidine-tagged *P1*Doc* and *P1*Doc^H66Y^. For non-toxic *P1*Doc^H66Y^, a clear signal can be detected on western blot four hours after induction, suggesting that significant overexpression is obtained (Figure 7). For *P1*Doc* on the other hand, no signal was obtained in western blot. Most likely, this is caused by the activity of the protein itself: by directly inhibiting translation, it inhibits its own production. Given that *P1*Doc is an enzyme, even a few molecules are likely sufficient to shut down the translation machinery. When monitoring the growth of *E. coli* EPI400 cells containing pJYP2_doc* and pJYP2_*doc*^H66Y^, induction of expression via IPTG, theophylline and CopyCutter Induction Solution did not affect the growth of cells harboring plasmids encoding *P1*Doc^H66Y^, and also the growth of non-induced cells harboring *P1*Doc* was not affected (Figure S1). In contrast, upon induction of *P1*Doc*, growth initially slowed down but caught up afterwards, likely due to mutations in doc, as we also observed in the spot tests (Figure S1).

**Figure 8:**
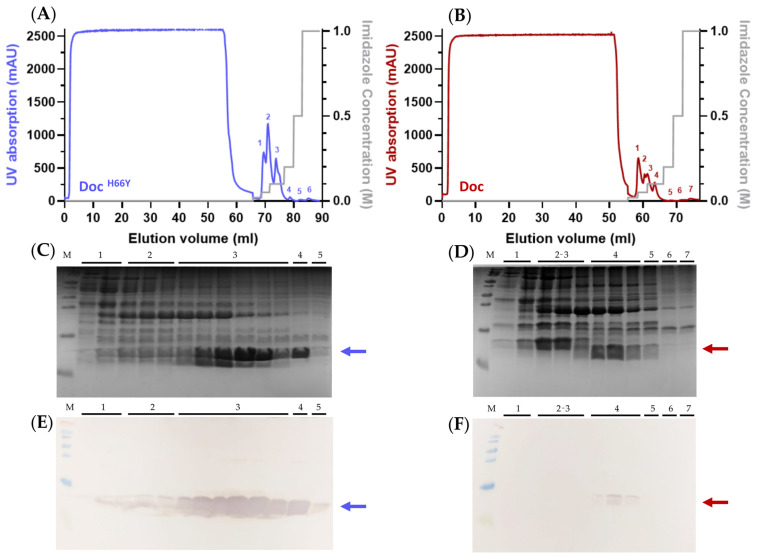
IMAC purification of *P1*Doc^H66Y^ (blue) and *P1*Doc* (red) using the P*tac*—RS_theo_ strategy. Fractions collected during IMAC elution (**A**,**B**) were analyzed through 20% SDS-PAGE (**C**,**D**) and anti-histidine Western blot (**E,F**). The blue and red arrows respectively indicate *P1*Doc^H66Y^ and *P1*Doc* (14.6 kDa).

To assess the potential of the P*tac*—RS_theo_ system for protein production, large-scale overexpression of his-tagged P1Doc and P1Doc^H66Y^ was attempted. Using material from larger volumes allowed western blot signals to be obtained for both *P1*Doc* and *P1*Doc^H66Y^. The signal for *P1*Doc* remained nonetheless significantly weaker than that for the non-toxic variant (Figure 8). Hence, overexpression and purification in reasonable amounts are difficult when the protein of interest directly targets the transcription or translation machinery. Consequently, our approach is still most useful for cloning particularly potent toxins and performing in vivo activity assays but less so for producing protein for biochemical or biophysical studies.

## 3. Conclusions

We evaluated different controlling systems for the cloning of highly toxic genes, minimizing leaky expression to almost zero. It must be noted that this is the first time that the highly toxic barnase and *Vc*ParE2 were cloned in the absence of their cognate inhibitors barstar and *Vc*ParD2, respectively. However, for overexpression and production purposes of the toxin protein, the yields remain low. However, with the evolving techniques, the amount might be sufficient for downstream study experiments on the toxin protein. Although this system was evaluated for toxins for which antitoxins exist, the antitoxins are not needed in this strategy, allowing the expansion of this strategy for all toxins. We designed a model system to efficiently clone toxic or unstable genes which might be optimized to overexpress and produce toxic/unstable proteins in the future.

## 4. Materials and Methods

### 4.1. Cloning and Transformation

#### 4.1.1. P*fdeA* Promoter

The pET22b expression vector was modified by inserting the FdeR transcriptional activator and promoter–operator region (P*fdeA*—*fdeO*) followed by a vitamin B_12_ riboswitch (RS_B12_) into the HpaI and XhoI site (Table S7). The resulting pJYP1 (Accession Number: OQ725380) vector contains a BglII restriction site downstream of the riboswitch to allow toxin cloning. Synthetic genes coding for different toxins were obtained via Twist Bioscience (San Francisco, CA, USA). The primers used for amplification of the different toxins prior to cloning are listed in Table 2. A polyhistidine-tag was placed N- or C-terminally, based on known purification strategies for the respective toxins (Table 3). The lyophilized synthetic DNA was dissolved to a concentration of 20 ng/μL. Using HiFi DNA assembly (New England Biolabs, Ipswich, MA, USA), all toxin genes were cloned in pJYP1 digested with BglII, resulting in different pJYP1_toxin vectors. CaCl_2_-competent EPI400 *E. coli* cells (*mcrA ∆(mrr-hsdRMS-mcrBC) φ80dlacZ∆M15 ∆lacX74 recA1 endA1 araD139 ∆(ara, leu)7697 galU galK λ–rpsL nupG tonA ∆pcnB dhfr*) were then transformed with the assembled DNA [39]. The competent cells were thawed on ice before adding 2 μL of the assembled DNA mixture. After 30 min incubation on ice, a heat shock was performed at 42 °C for 1 min. The transformation mixture was diluted by adding 1 mL of LB medium *(Bertani, 1951)* supplemented with 50 nM vitamin B_12_ after which it was incubated while shaking at 37 °C for 4 h to allow phenotypic expression. Hundred μL of the culture was spread on an LB plate supplemented with 100 μg/mL of ampicillin and 50 nM of vitamin B_12_ before overnight incubation at 37 °C. Transformants were first tested for the presence of an intact toxin by comparing the growth of the colonies on both LB agar plates supplemented with 100 µg/mL ampicillin and 50 nM of vitamin B_12_ and LB agar plates supplemented with 100 µg/mL ampicillin and 100 µg/mL naringenin as inducer. The colonies that showed reduced growth for the latter conditions were further screened through sequencing from miniprepped plasmids. The plasmids were recovered by preparing an overnight culture of *E. coli* EPI400 (pJYP1_toxin) in LB supplemented with 100 μg/mL of ampicillin and 50 nM vitamin B_12_. This was used to inoculate 10 mL of fresh LB with 100 μg/mL of ampicillin, 1× CopyCutter Induction Solution (LGC Biosearch Technologies, Teddington, England) and 50 nM vitamin B_12_, which was added to reach an OD_600_ of 0.2. This culture was incubated for 4 h at 37 °C shaking, after which plasmid DNA was purified following the Monarch^®^ Plasmid Miniprep Kit (NEB, Ipswich, MA, USA). Sequencing was performed using primers Seq_Primer25 (5′-ttgagcctggccatgacaac-3′) and Seq_Primer26 (5′-ccagcctacacgggagagtg-3′).

#### 4.1.2. Tac Promoter

Restriction digestion with BglII and XhoI (NEB) for 1 h at 37 °C was performed to remove the T7 promoter and lac operator of vector pET22b. Synthetic DNA fragments comprising the tac promoter (P*tac*) and lac operator followed by the theophylline (RS_theo_) or vitamin B_12_ (RS_B12_) riboswitch upstream of a C- or N-terminal his-tagged toxin gene and 25 bp overlap with the pET22b vector for Gibson assembly cloning were designed (Table 3 and Table S7) and ordered via Twist Bioscience (San Francisco, CA, USA). Gibson assembly was performed to insert the construct at the BglII and XhoI restriction sites of vector pET22b, resulting in the pJYP2_toxin and pJYP3_toxin constructs, for the theophylline and vitamin B_12_ riboswitch, respectively. The assembled DNA was subsequently transformed into CaCl_2_-competent *E. coli* EPI400 cells, as described above. An LB agar plate supplemented with 100 µg/mL ampicillin was plated with 100 µL of the transformation product and incubated overnight at 37 °C. A first screening for positive transformants was performed by comparing the growth of the colonies on both LB agar plates supplemented with 100 µg/mL ampicillin and LB agar plates supplemented with 100 µg/mL ampicillin, 0.5 mM IPTG and 2 mM theophylline (60 mg/mL stock dissolved in 200 mM NaOH). The colonies that showed reduced growth for the latter conditions were further screened by colony PCR and sequencing, using primers Seq_Primer37 (5′-gatcttccccatcggtgatg-3′) and Seq_Primer38 (5′-gcagcagccaactcagcttc-3′).

#### 4.1.3. Arabinose Promoter

Analogously to the *tac* system, a synthetic construct containing *araC* and the C-terminally his-tagged *Vibrio cholerae parE2* toxin gene under the control of P*_BAD_* in combination with the theophylline riboswitch (Table 3 and Table S7) was cloned in the pET22b vector digested with BglII/XhoI.

### 4.2. Spot Test

An overnight culture of the positive transformants was prepared in 5 mL of LB medium supplemented with 100 µg/mL ampicillin (and 50 nM of vitamin B_12_ for the RS_B12_ constructs). The next day, a serial dilution of the overnight culture was made in LB medium (10^0^–10^−6^). Five µL of each dilution was spotted on square 50 mL LB plates supplemented with 100 µg/mL ampicillin (non-induced) and on LB agar plates supplemented with 100 µg/mL ampicillin, 2 mM theophylline and 0.5 mM IPTG (induced) or either one of each inducing agent (partly induced) for the P*tac*—RS_theo_ strategy. For the P*fdeA*—RS_B12_ strategy, spots were placed on LB agar plates supplemented with 100 µg/mL ampicillin and 50 nM vitamin B_12_ (non-induced) and LB agar plates supplemented with 100 µg/mL ampicillin and 100 µg/mL naringenin (induced), as well as LB agar plates with only 100 µg/mL ampicillin, or with both 50 nM vitamin B_12_ and 100 µg/mL naringenin (partly induced).

### 4.3. Plate Reader Experiment

Overnight cultures were prepared for *E. coli* EPI400 pJYP2_doc* and pJYP2_*doc*^H66Y^ in 5 mL LB medium supplemented with 100 µg/mL ampicillin. Cultures were brought to an OD of 2. A 96-well plate was prepared by adding 2 µL of the cultures and 178 µL of LB ampicillin medium. The OD_600_ was measured every 20 min using the Biotek Synergy H1 (BioSPX, Abcoude, The Netherlands) microplate reader while shaking at 425 cpm—37 °C. Once an OD of approximately 0.2 was measured, 2 mM theophylline, 0.5 mM IPTG and 1× CopyCutter^TM^ Induction Solution was added for the induced samples, and an equal volume of LB medium was added for the non-induced samples. Cultures were further grown in the same conditions for a total of 16 h.

### 4.4. Expression Test

Expression was first tested on a small scale. Individual colonies of *E. coli* EPI400 carrying the pJYP2_doc* and pJYP2_*doc*^H66Y^ plasmid were picked to start an overnight preculture of 10 mL of LB enriched with 100 µg/mL ampicillin at 37 °C. A main culture of 80 mL of LB supplemented with 100 µg/mL ampicillin, 1× CopyCutter^TM^ Induction Solution and a 20-fold dilution of the preculture was grown until OD_600_ = 1. Next, protein expression was induced by adding 0.5 mM IPTG and 2 mM theophylline. Samples of 10 mL collected before and 4 h after induction were centrifuged for 9 min at 2500× g, and the pelletized cells were resuspended in 200 µL B-PER^TM^ bacterial protein extraction reagent (ThermoFisher, Waltham, MA, USA) After 10 min of incubation, the cell lysis product was centrifuged for 5 min at 17,000× g. The resulting supernatant was used for SDS-PAGE and anti-his-tag Western blot analysis [40,41]. For western blot, proteins were transferred from the SDS-PAGE gel to a blotting membrane (Trans-Blot Turbo Transfer System, Bio-Rad, Hercules, CA, USA) by applying 25V for 5 min. The membrane was blocked for 45 min in 5% milk powder in 1X TBS, followed by a 1 h incubation with 10 µL mouse anti-his (Bio-Rad) in 10 mL 5% milk in 1X TBS. After washing the membrane with 1X TBS, a second incubation step of 40 min was performed with goat-anti-mouse IgG (Merck, Darmstadt, Germany) in 10 mL 5% milk in 1X TBS. Subsequently, the membrane was washed 3 times with 1X TBS, and protein visualization was obtained by a final 15 min incubation of the membrane in 10 mL of western blot buffer (100 mM Tris, 50 mM MgCl2, 100 mM NaCl) combined with 50 µL NBT-BCIP substrate.

For large-scale protein purification, an overnight preculture containing 250 mL of LB, 100 µg/mL ampicillin and a picked colony was grown at 37 °C. A 50-fold dilution of this preculture was used to inoculate 12 L of the main culture (12X 1 L LB enriched with 100 mg/mL ampicillin and 1× CopyCutter^TM^ Induction Solution). The main culture was grown at 37 °C until OD_600_ = 0.8, after which 0.5 mM IPTG and 2 mM theophylline were added to induce protein expression. Cultures were grown for 4 h at 28 °C, and cells were harvested by centrifugation for 15 min 6200 g. The resulting pellet was dissolved in 20 mM Tris pH 8, 500 mM NaCl supplemented with a protease inhibitor (cOmplete^TM^ ULTA tablets, EDTA free, Roche, Basel, Switzerland), flash-frozen using liquid nitrogen and stored at −80 °C.

### 4.5. qPCR

Similar to Anindyajati et al., qPCR was performed to determine the primer efficiency [42]. Primer sets that target (1) the origin of replication (*ori*) in pET22b and (2) the d-1-deoxyxylulose 5-phosphate synthase gene (*dxs*) in the *E. coli* chromosome (Table 4) were used for qPCR on two technical replicates of the template with concentrations ranging from 0.00002 to 0.02 ng/µL (pET22b) and 0.00002 to 2 ng (genomic DNA). EPI400 *E. coli* chromosome was isolated with the genomic DNA preparation kit (Qiagen, Hilden, Germany), and pET22b was extracted using the Monarch^®^ Plasmid Miniprep Kit (NEB, Ipswich, MA, USA). Primer efficiency (E) was determined by applying equation 1 after fitting a regression curve through the obtained Ct values as a function of the log of the starting quantity.

(1) $$E=10^{-1/slope}$$

Cell lysates from four overnight cultures of EPI400 *E. coli* cells carrying pJYP2-doc* and pJYP2-*doc*^H66Y^ grown in the absence (non-induced) and presence (induced) of 1× CopyCutter Induction Solution were extracted as follows: 1 mL of overnight cell culture at OD_600_ = 2 was centrifuged for 1 minute at 16,000× *g*. Hereafter, the pellet was resuspended in 1 mL of milli-Q water, incubated at 95 °C for 10 min and again centrifuged at 16,000× *g* for 2 minutes. The resulting supernatant was collected, and a 10-fold dilution was prepared in milli-Q water for qPCR. A 1 µL volume of each 10-fold dilution was combined with 2× GoTaq qPCR Master Mix (Promega) and both *ori* and *dxs* primers in separate reactions. For each condition, 6 replicates and 2 negative controls were subjected to the following amplification: 5.5 min at 95 °C, 40 cycles consisting of 10 s denaturation at 95 °C and 30 s annealing at 55 °C.

To gain an idea of how much more plasmid is present in the induced state compared to the non-induced state, the ratio of plasmid to chromosome per cell (P/C) was calculated by applying following equation:

(2) $$\frac{Number\;of\;plasmid}{Number\;of\;chromosome}=\frac{{E_{C}}^{{Ct}_{C}}}{{E_{P}}^{{Ct}_{P}}}$$

Here, E_C_ represents the primer efficiency of the primer set targeting the chromosome (*dxs*), Ct_C_ the threshold value measured with the *dxs* primers, E_P_ the primer efficiency of the primer set targeting the plasmid (*ori*) and Ct_P_ the threshold value measured with the *ori* primers. To quantify the effect of induction with CopyCutter Induction Solution, P/C values from the induced state were divided by the ones from the non-induced state for pJYP2_doc* and pJYP2_*doc^H66Y^*.

### 4.6. Protein Purification

The resuspended pellets from 12 L of cell culture were thawed and DNase I (50 µg/mL) and MgCl*_2_*(5 mM) were added. Cells were lysed using the cell cracker at 20 KPSI, and the resulting cell lysate was centrifuged at 46,000× *g*. The supernatant was filtered through a 0.45 µm filter and loaded on a 1 mL HisTrap HP column (Cytiva, Marlborough, MA, USA) equilibrated with 20 mM Tris pH 8, 500 mM NaCl. A step gradient of 20 mM Tris pH 8, 500 mM NaCl, 1 M imidazole (0–2–5–10–20–50–100%, 3 column volumes each) was applied. The progress of purification was visualized with SDS-PAGE and anti-his-tag Western blot.

## Figures and Tables

**Table 1:** Overview of toxins cloned in four different gene expression systems. +: mutation-free clone; -: no clone; ~ : toxic clone with mutation; NT: not tested.

Toxins	Accession Number Uniprot	P*fdeA*—RS_B12_	P*tac*—RS_B12_	P*tac*—RS_theo_	P*_BAD_*—RS_theo_
P1Doc	Q06259 DOC_BPP1	+	-	~	NT
P1DocH66Y	/	+	+	+	NT
EcMazF	P0AE70 MAZF_ECOLI	+	+	+	NT
EcMazFE24A	/	+	+	+	NT
EcParE2	A0A0H3JHG3 A0A0H3JHG3_ECO57	+	-	+	NT
FCcdB	P62554 CCDB_ECOLI	+	-	~	NT
VcHigB2	Q9KMA6·HIGB2_VIBCH	+	-	+	NT
barnase	P00648 RNBR_BACAM	+	-	~	NT
VcParE2	Q9KMJ0 Q9KMJ0_VIBCH	-	-	-	+

**Table 2:** Primers used for Gibson assembly of toxins in pJYP1.

Toxin	Primer	Sequence
*Ec*MazF-His	MazFHis_F1	5′-ccttcttctattgtggatgctttacaatggtaagccgatacgtacccg-3′
	MazFHis_R1	5′-tccccactcgccagatttacgaagatcagtgatgatgatgatgatggctgc-3′
His-barnase	Hisbarnase_F1	5′-ccttcttctattgtggatgctttacaatgggcagcagccatcacc-3′
	Hisbarnase_R1	5′-tccccactcgccagatttacgaagagatctttatctgatttttgtaaaggtctgataatggtccg-3′
barnase-His	barnaseHis_F1	5′-ccttcttctattgtggatgctttacaatggcacaggttatcaacacgtttgacgg-3′
	barnaseHis_R1	5′-tccccactcgccagatttacgaagatcagtgatgatgatgatgatggctgc-3′
His-*Vc*HigB2	HisHigB2_F1	5′-ccttcttctattgtggatgctttacaatgggcagcagccatcacc-3′
	HisHigB2_R1	5′-tccccactcgccagatttacgaagatcacgattgctcattgcgc-3′
*Vc*ParE2-His	*Vc*ParE2His_F1	5′-ccttcttctattgtggatgctttacaatgaaaccatttaatcttaccgtcgccgc-3′
	*Vc*ParE2His_R1	5′-tccccactcgccagatttacgaagatcagtgatgatgatgatgatgtgcg-3′
*P1*Doc-His	DocHis_F1	5′-ccttcttctattgtggatgctttacaatgaggcatatatcaccggaagaac-3′
	DocHis_R1	5′-tccccactcgccagatttacgaagatcagtgatgatgatgatgatggctgc-3′
*Ec*ParE2-His	*Ec*ParE2His_F1	5′-ccttcttctattgtggatgctttacaatgttacccgtgttatggcttgaaagcgc-3′
	*Ec*ParE2His_R1	5′-tccccactcgccagatttacgaagatcagtgatgatgatgatgatggctgc-3′
*F*CcdB	CcdB_F1 CcdB_R1	5′-cttcttctattgtggatgctttacaatgcagtttaaggtttacacctata-3′ 5′-cactcgccagatttacgaagagatcttatattccccagaacatcaggtta-3′

**Table 3:** Plasmids used in this study. Toxins cloned in pJYP1 are *P1*Doc, *P1*Doc^H66Y^, *Ec*MazF, *Ec*MazF^E24A^, *Ec*ParE2, *Vc*HigB2, barnase and *F*CcdB. For pJYP2: *P1*Doc*, *P1*Doc^H66Y^, *Ec*MazF, *Ec*MazF^E24A^, *Ec*ParE2, *Vc*HigB2, barnase* and *F*CcdB*. For pJYP3: *P1*Doc^H66Y^, *Ec*MazF and *Ec*MazF^E24A^.

Plasmids	Description
pJYP1_toxin	FdeR—P*fdeA*—RS_B12_—toxin in HpaI/XhoI site of pET22b
pJYP2_toxin	LacI—P*tac*—RS_theo_—toxin in BglII/XhoI site of pET22b
pJYP3_toxin	LacI—P*tac*—RS_B12_—toxin in BglII/XhoI site of pET22b
pJYP4_*Vc*ParE2	AraC—P*_BAD_*—RS_theo_—*Vc*ParE2 in BglII/XhoI site of pET22b

**Table 4:** Plasmid specific (*ori*) and genome specific (*dxs*) primer sets used for qPCR.

Primer Name	Primer Sequence	Primer Length	Product Size	Source
ori.FW	ATACCTGTCCGCCTTTCTCC	20 nt	86 bp	[42]
ori.RV	GAACGACCTACACCGAACTGAG	22 nt	86 bp	[42]
dxs.FW	CGAGAAACTGGCGATCCTTA	20 nt	113 bp	[43]
dxs.RV	CTTCATCAAGCGGTTTCACA	20 nt	113 bp	[43]

## Data Availability

The pJYP1 plasmid sequence is submitted to GenBank with Accession Number OQ725380 and Addgene (Addgene Plasmid #202406).

## Supplementary Materials

The following supporting information can be downloaded at: https://www.mdpi.com/article/10.3390/toxins15080508/s1, Table S1: overview of toxicity levels for the P*fdeA*-RS_B12_ strategy; Table S2: overview of toxicity levels for the P*tac*-RS_theo_ strategy; Figure S1: OD profile of *E. coli* EPI400 CopyCutter cells carrying either the plasmids pJYP1_*doc* or pJYP2_*doc*^H66Y^; Supplementary qPCR results containing Figure S2: qPCR result for determination of plasmid efficiency; Table S3: PCR efficiency values (E) for the plasmid-specific *ori* primers and the genome-specific *dxs* primers; Table S4: Threshold cycle (Ct) values determined by qPCR using (**A**) the plasmid-specific *ori* primers and (**B**) the genome specific *dxs* primers; Table S5: P/C values calculated from qPCR performed on the cell lysate of *E. coli* EPI400 cells that carry pJYP2_*doc* and pJYP2_*doc*^H66Y^ in non-induced state (NI) and induced with CCIS (I); Table S6: Ratio of the Doc and Doc^H66Y^ P/C values in induced and non-induced state; Table S7: DNA sequences of the synthetic constructs.
