# Supplementary material for: A Multi-Layer-Controlled Strategy for Cloning and Expression of Toxin Genes in Escherichia coli
Source: Toxins (Basel). 2023 Aug 18;15(8):508. doi: 10.3390/toxins15080508 (PMC10467106; doi:10.3390/toxins15080508)
Supplement: Supplementary file 1 [file toxins-15-00508-s001.zip › toxins-2357833-supplementary.pdf]

A Multi-Layer-Controlled Strategy for Cloning and Expression of Toxin Genes in *Escherichia coli*

Table S1. Overview of toxicity levels for the P*fdeA* – RS_{B12} strategy (expressed in CFU/mL) in presence and absence of vitamin B_{12} (B_{12}) and naringenin (nar). Colonies are counted from the spot test on different LB ampicillin plates for *E. coli* EPI400 harboring toxins *P1*Doc, *P1*Doc^{H66Y}, *Ec*MazF, *Ec*$MazF^{E24A}$, *Ec*ParE2, *Vc*HigB2, barnase and *F*CcdB.

Toxin	B_{12} (CFU/mL)	nar (CFU/mL)	B_{12} + nar (CFU/mL)	/ (CFU/mL)
*P1*Doc	0.8×10^9	1.0×10^5	2.0×10^5	0.6×10^9
*P1*Doc^{H66Y}	1.2×10^9	0.6×10^9	0.2×10^9	0.6×10^9
*Ec*MazF	0.8×10^9	0.8×10^9	0.5×10^9	0.6×10^9
Ec$MazF^{E24A}$	0.6×10^9	0.6×10^9	1.0×10^9	0.2×10^9
*Ec*ParE2	1.0×10^9	0.8×10^9	1.0×10^9	1.2×10^9
*Vc*HigB2	1.0×10^9	0.8×10^5	0.6×10^5	1.0×10^9
barnase	0.4×10^9	1.0×10^5	2.0×10^5	0.6×10^9
*F*CcdB	0.4×10^8	0.4×10^5	1.0×10^6	0.8×10^8

Table S2. Overview of toxicity levels for the P*tac* – RS_{theo} strategy (expressed in CFU/mL) in presence and absence of IPTG and theophylline (theo). Colonies are counted from the spot test on different LB ampicillin plates for *E. coli* EPI400 harboring toxins *P1*Doc*, *P1*Doc^{H66Y}, *Ec*MazF, *Ec*$MazF^{E24A}$, *Ec*ParE2, *Vc*HigB2, barnase* and *F*CcdB*.

Toxin	/ (CFU/mL)	theo + IPTG (CFU/mL)	IPTG (CFU/mL)	theo (CFU/mL)
*P1*Doc*	0.8×10^9	0.3×10^5	0.2×10^8	1.0×10^9
*P1*Doc^{H66Y}	1.4×10^9	0.4×10^8	0.8×10^9	0.4×10^9
*Ec*MazF	0.2×10^9	0.8×10^5	0.8×10^7	1.4×10^7
Ec$MazF^{E24A}$	0.6×10^9	$<0.2 \times 10^6$	1.6×10^9	0.8×10^9
*Ec*ParE2	0.8×10^8	0.4×10^7	0.6×10^8	0.8×10^8
*Vc*HigB2	1.4×10^8	$<0.2 \times 10^4$	0.4×10^5	0.4×10^5
barnase*	0.4×10^9	$<0.2 \times 10^4$	0.2×10^6	0.2×10^5
*F*CcdB*	0.3×10^9	0.3×10^6	0.4×10^7	0.2×10^7

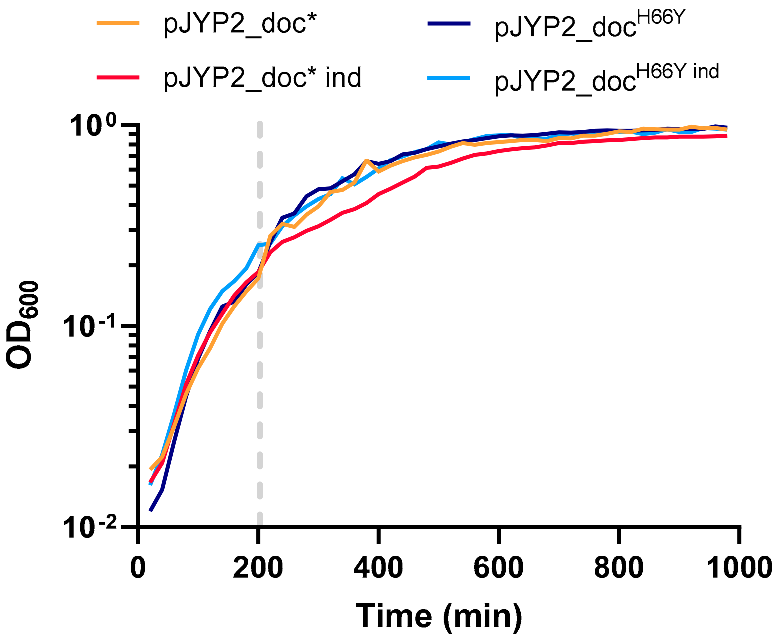

Figure S1. OD profile of *E. coli* EPI400 CopyCutter cells carrying either the plasmid pJYP2_*doc** or pJYP2_*doc*^{H66Y}. The vertical line shows the moment where cells harbouring pJYP2_*doc** (red) and pJYP2_*doc*^{H66Y} (light blue) were induced with CCIS, IPTG and theophylline. For non-induced pJYP2_*doc** (orange) and pJYP2_*doc*^{H66Y} (dark blue) cells, LB medium was added instead.

Supplementary qPCR results.

Primer efficiency was calculated for primer sets that target (1) the origin of replication (*ori*) in pET22b and (2) the d-1-deoxyxylulose 5-phosphate synthase gene (*dxs*) in the *E. coli* chromosome. Standard curves and primer efficiency (E) determined by qPCR are shown in (Figure S1) and (Table S1) respectively.

A

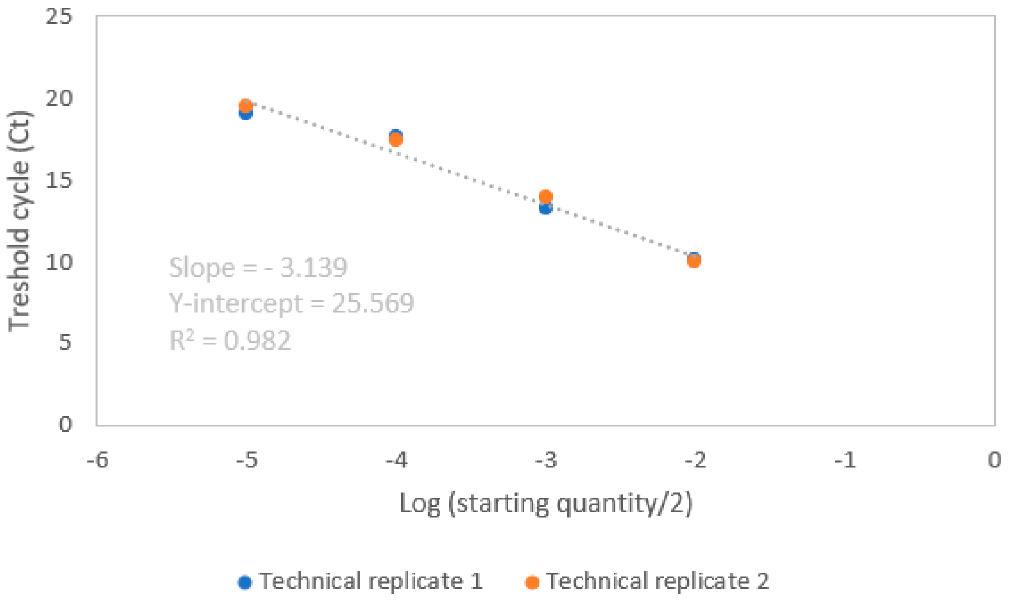

B

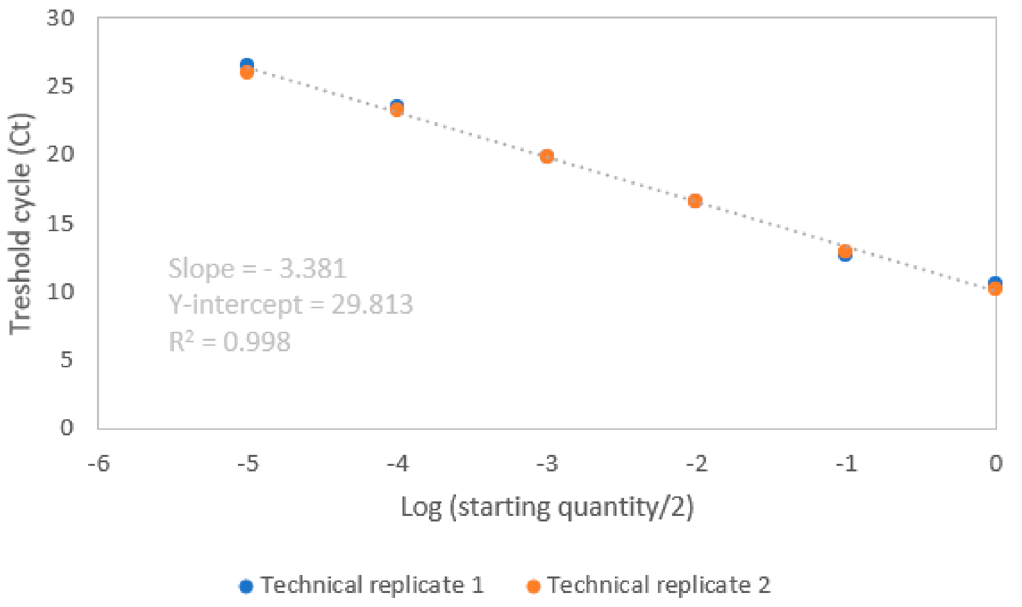

Figure S2. qPCR result for determination of plasmid efficiency. Graphs A and B show the correlation between Ct and the log of the starting quantity divided by two when performing a qPCR with plasmid specific *ori* primers and genome specific *dxs* primers respectively. The best fit and corresponding slope, Y-intercept and R^2 value are indicated in grey.

Table S3. PCR efficiency values (E) for the plasmid-specific *ori* primers and the genome-specific *dxs* primers. A primer efficiency of 2.00 corresponds with 100% amplification efficiency.

Primers	PCR efficiency
ori primers	2.082
dxs primers	1.976

Using these primers, qPCR was performed with as template the cell lysates from four overnight cultures of EPI400 *E. coli* cells carrying pJYP2_*doc* and pJYP2_*doc*^{H66Y} grown in absence (non-induced) and presence (induced) of 1x CopyCutter Induction Solution (CCIS). Looking at the raw data, Ct values are similar for amplification of genomic DNA in cell lysates extracted from precultures grown with or without CCIS (Table S2B). However, for amplification of plasmid DNA present in these cell lysates a clear difference in Ct values is observed (Table S2A). The Ct values are lower for cell lysates of precultures grown in presence of CCIS, indicating that more plasmid is present. When calculating the P/C values (Table S3) and the ratio of the Doc and DocH66Y P/C values in induced and non-induced state (Table S4), we see that in both cases the number of plasmids increases by a factor of approximately 50 after induction with CCIS. We want to include the sidenote that the values obtained by qPCR are not completely accurate. This 50-fold increase might be an overestimation. However, it clearly shows a significant increase in plasmid copy number. This emphasized the relevance of using the CopyCutter EPI400 strain.

Table S4. Threshold cycle (Ct) values determined by qPCR using (**A**) the plasmid-specific *ori* primers and (**B**) the genome specific *dxs* primers. Crossed out values are not included in the calculation of the average Ct value. Templates used for qPCR are the cell lysates of EPI400 *E. coli* cells harbouring pJYP2*doc* and pJYP2_*doc*^{H66Y} grown in absence (NI) and presence (I) of 1x CopyCutter Induction Solution. For each condition, Ct values were measured for three biological replicates (1-3) with each two technical replicates (a,b). When N/A is written, the required fluorescent threshold was not reached after 40 PCR cycli.

A

	$Ct_{,1a}$	$Ct_{,1b}$	$Ct_{,2a}$	$Ct_{,2b}$	$Ct_{,3a}$	$Ct_{,3b}$	Neg_a	Neg_b	$Ct_{,AVG}$ ± STDEV
Doc $_{NI}$	25.24	24.68	23.57	23.95	22.69	~~32.63~~	31.30	30.04	24.03 ± 0.99
Doc $_{I}$	18.33	18.53	18.49	18.18	18.79	18.70	31.97	30.51	18.51 ± 0.23
$Doc^{H66Y}{}_{NI}$	24.04	24.15	23.21	22.83	24.67	23.73	32.32	36.32	23.77 ± 0.67
$Doc^{H66Y}{}_{I}$	17.96	19.07	18.91	18.96	17.97	17.74	32.25	27.73	18.44 ± 0.61

B

	$Ct_{,1a}$	$Ct_{,1b}$	$Ct_{,2a}$	$Ct_{,2b}$	$Ct_{,3a}$	$Ct_{,3b}$	Neg_a	Neg_b	$Ct_{,AVG}$ ± STDEV
Doc $_{NI}$	24.30	24.69	24.54	24.18	24.14	~~N/A~~	36.83	37.57	24.37 ± 0.24
Doc $_{I}$	24.04	24.52	24.35	23.97	24.41	24.24	N/A	N/A	24.25 ± 0.21
$Doc^{H66Y}{}_{NI}$	25.94	25.27	24.19	25.00	23.79	24.83	37.48	N/A	24.84 ± 0.77
$Doc^{H66Y}{}_{I}$	24.65	24.79	25.15	24.97	24.61	24.69	N/A	38.11	24.81 ± 0.21

Table S5 P/C values calculated from qPCR performed on the cell lysate of *E. coli* EPI400 cells that carry pJYP2_*doc* and pJYP2_*doc*^{H66Y} in non-induced state (NI) and induced with CCIS (I).

	P/C value
Doc $_{NI}$	0.358
Doc $_{I}$	18.984
$Doc^{H66Y}{}_{NI}$	0.592
$Doc^{H66Y}{}_{I}$	29.173

Table S6. Ratio of the Doc and Doc^{H66Y} P/C values in induced and non-induced state.

	**$(P/C)_{I}$ / $(P/C)_{NI}$ value**
Doc	53.0
Doc^{H66Y}	49.2

Table S7. DNA sequences of the synthetic constructs for Gibson Assembly insertion in XhoI/BglII-restricted pET22b. Following regions are indicated: (1) P*tac* or P_{BAD} promoter in bold, (2) vitamin B12 or theophylline riboswitch (grey background) and (3) his-tagged toxin protein (start and stop codon underlined). *P1*Doc is used as example for the P*tac* – RS_{B12} and the P*tac* – RS_{theo} systems, *Vc*ParE2 as example for P_{BAD} – RS_{theo}), (4) *araC* activator in small letters. The *lacI* repressor is not included in the synthetic fragment since it is already present in the backbone of the pET22b vector.

**Strategy**	**Synthetic construct**
P*tac* – RS_{B12}	CGATGCGTCCGGCGTAGAGGATCGATTAATGTGAGTTAGCTCACTCATTAGGCACCC<br>CAGGC**TTGACAATTAATCATCGGCTCGTATAATG**TGTGGAATTGTGAGCGGATAAC<br>AATTTCACACGCCGGTCCTGTGAGTTAATAGGGAATCCAGTGCGAATCTGGAGCTGA<br>CGCGCAGCGGTAAGGAAAGGTGCGATGATTGCGTTATGCGGACACTGCCATTCGGT<br>GGGAAGTCATCATCTCTTAGTATCTTAGATACCCCTCCAAGCCCGAAGACCTGCCGG<br>CCAACGTCGCATCTGGTTCTCATCATCGCGTAATATTGATGAAACCTGCGGCATCCTT<br>CTTCTATTGTGGATGCTTTACAATGAGGCATATATCACCGGAAGAACTTATTGCGCTT<br>CATGATGCGAATATAAGCCGCTACGGCGGCCTGCCGGGAATGTCTGATCCGGGTAG<br>GGCAGAGGCCATTATCGGGAGAGTTCAGGCCAGAGTTGCCTACGAAGAGATCACCG<br>ACCTTTTCGAAGTCTCCGCCACCTACCTGGTGGCTACAGCGAGAGGGCATATATTCA<br>ATGATGCCAATAAGCGTACCGCGCTAAACAGTGCGCTGCTATTTCTACGCCGTAACG<br>GGGTGCAGGTATTTGATTCACCTGAACTGGCAGACCTTACTGTAGGAGCTGCGACTG<br>GCGAGATATCTGTATCTTCTGTCGCCGACACGTTACGTAGATTGTATGGTTCTGCGGA<br>GGGCAGCAGCCATCATCATCATCATCACTGATCGAGCACCACCACCACCACCACTG<br>AGATC
P*tac* – RS_{theo}	CGATGCGTCCGGCGTAGAGGATCGATTAATGTGAGTTAGCTCACTCATTAGGCACCC<br>CAGGC**TTGACAATTAATCATCGGCTCGTATAATG**TGTGGAATTGTGAGCGGATAAC<br>AATTTCACACGGTGATACCAGCATCGTCTTGATGCCCTTGGCAGCACCCTGCTAAGG<br>AGGTAACAACAAGATGAGGCATATATCACCGGAAGAACTTATTGCGCTTCATGATG<br>CGAATATAAGCCGCTACGGCGGCCTGCCGGGAATGTCTGATCCGGGTAGGGCAGAG<br>GCCATTATCGGGAGAGTTCAGGCCAGAGTTGCCTACGAAGAGATCACCGACCTTTTC<br>GAAGTCTCCGCCACCTACCTGGTGGCTACAGCGAGAGGGCATATATTCAATGATGCC<br>AATAAGCGTACCGCGCTAAACAGTGCGCTGCTATTTCTACGCCGTAACGGGGTGCA<br>GGTATTTGATTCACCTGAACTGGCAGACCTTACTGTAGGAGCTGCGACTGGCGAGAT<br>ATCTGTATCTTCTGTCGCCGACACGTTACGTAGATTGTATGGTTCTGCGGAGGGCAGC<br>AGCCATCATCATCATCATCACTGATCGAGCACCACCACCACCACCACTGAGATC
P_{BAD} – RS_{theo}	TGCGTCCGGCGTAGAGGATCGATCGAAGATCTttatgacaacttgacggctacatcattcactttttcttcaca<br>accggcacggaactcgctcgggctggccccggtgcatttttaaatacccgcgagaaatagagttgatcgtcaaaaccaacattgcga<br>ccgacggtggcgataggcatccgggtggtgctcaaaagcagcttcgcctggctgatacgttggtcctcgcgccagcttaagacgctaa<br>tccctaactgctggcggaaaagatgtgacagacgcgacggcgacaagcaaacatgctgtgcgacgctggcgatatcaaaattgctgt<br>ctgccaggtgatcgctgatgtactgacaagcctcgcgtacccgattatccatcggtggatggagcgactcgttaatcgcttccatgcgcc<br>gcagtaacaattgctcaagcagatttatcgccagcagctccgaatagcgcccttccccttgcccggcgttaatgatttgcccaaacaggt<br>cgctgaaatgcggctggtgcgcttcatccgggcgaaagaaccccgtattggcaaatattgacggccagttaagccattcatgccagta<br>ggcgcgcggacgaaagtaaacccactggtgataccattcgcgagcctccggatgacgaccgtagtgatgaatctctcctggcgggaa

	cagcaaaatatcacccggtcggcaaacaaattctcgtccctgatttttcaccacccccctgaccgcgaatggtgagattgagaatataacc<br>tttcattcccagcggtcggtcgataaaaaaatcgagataaccgttggcctcaatcggcgttaaacccgccaccagatgggcattaaacg<br>agtatcccggcagcaggggatcattttgcgcttcagccatACTTTTCATACTCCCGCCATTCAGAG**AAGAA**<br>**ACCAATTGTCCATATTGCATCAGACATTGCCGTCACTGCGTCTTTTACTGGCTCTT**<br>**CTCGCTAACCAAACCGGTAACCCCGCTTATTAAAAGCATTCTGTAACAAAGCGG**<br>**GACCAAAGCCATGACAAAAACGCGTAACAAAAGTGTCTATAATCACGGCAGAA**<br>**AAGTCCACATTGATTATTTGCACGGCGTCACACTTTGCTATGCCATAGCATTTTT**<br>**ATCCATAAGATTAGCGGATCCTACCTGACGCTTTTTATCGCAACTCTCTACTGTTT**<br>**CTCCAT**ACCCGTTTTTTGGGCTAACAGGAGGAATTAACGATATCGGTGATACCAGCA<br>TCGTCTTGATGCCCTTGGCAGCACCCTGCTAAGGAGGTAACAACAAGATGAAACCA<br>TTTAATCTTACCGTCGCCGCCAAAGCCGATTTACGTGATATTGCTTTATTCACTCAAC<br>GACGCTGGGGAAAAGAGCAGCGAAATGTTTATTTAAAGCAATTCGATGATTCCTTTT<br>GGCTTTTAGCGGAAAATCCCGACATTGGTAAATCATGCGATGAAATCCGAGAGGGA<br>TACAGAAAATTTCCCCAAGGGAGTCACGTCATCTTTTATCAGCAAACCGGCAGCCA<br>ACAAATCAGGGTGATCCGAATTCTTCATAAGAGCATGGATGTGAACCCAATATTCG<br>GCGCACATCATCATCATCATCACTGACTCGAGCACCACCACCACCACCACTGA
